# Three/four-dimensional (3D/4D) microscopic imaging and processing in clinical dental research

**DOI:** 10.1186/s12903-016-0282-0

**Published:** 2016-09-01

**Authors:** Ping Ye, Hong Yu, Mojgan Houshmandi

**Affiliations:** 1 Institute of Dental Research, Oral Health, Westmead Hospital, Westmead, Australia; 2 Affiliation of Faculty of Dentistry, the University of Sydney, Sydney, Australia; 3 Microscopy Laboratory, Westmead Institute for Medical Research, Westmead, Australia

**Keywords:** 3D reconstruction, 4D imaging, Live cell imaging, Carious lesion, Periodontitis, *Porphyromonas gingivalis*

## Abstract

**Background:**

Confocal laser scanning microscope (CLSM) has been widely employed in our laboratory for structural and functional analysis of clinical dental specimens and live cell imaging of cultured oral epithelial cells.

**Methods:**

In this vitro study, a Fluoview 1000 (Olympus) confocal system was utilised to study thick sections of carious lesions (40–100 μm) and periodontal disease tissue samples (20–40 μm) by 2D Z stacking imaging and 3-dimentional (3D) reconstruction. Four-dimensional (4D) imaging when including time or position points was used for live cells to assess penetration/localisation/co-localization of oral pathogen proteins and therapeutic drugs.

**Results:**

Three-dimensional (3D) reconstruction revealed latent features of carious hard tissues (strongly expressed amelogenin proteins in dentin tubules), and soft tissues (increased glial markers GFAP and S100B in pulp components). We also found the oral microbial specific pathogens, *Porphyromonas gingivalis* to be widely localised inside the periodontal pocket epithelial tissues as detected by 3D reconstruction from a series of 2D sections from periodontal disease tissue samples. 4D live cell imaging showed the diffusion patterns of fluorescent molecules in response to a bacterial virulence factor, the pathogen (gingipain haemagglutinin) domain that attacked epithelial integrity. This technology also showed uptake of a novel porphyrin-linked metronidazole antibiotic into epithelial cells to kill intracellular oral pathogen, *P. gingivalis*.

**Conclusions:**

Three/four-dimensional (3D/4D) imaging and processing in confocal microscopy is of great interest and benefit to clinical dental researchers.

**Electronic supplementary material:**

The online version of this article (doi:10.1186/s12903-016-0282-0) contains supplementary material, which is available to authorized users.

## Background

Confocal laser scanning microscope (CLSM) has been developed and improved enormously over the past 10 years. This powerful technology has several advantages over conventional epi-fluorescence microscopy, including improved performance in contrast and free out-of-focus blur for thin or thick specimens [1], excellent high resolution and analysis of fluorescent labelled thick specimens without physical sectioning [1, 2], capacity for complex three-dimensional (XYZ) architecture called 3D reconstruction [2], and 4D imaging and processing on live cells when including time (XYZT) or position (XYZP) points [3], and even 5D imaging when including numerous channels [3]. These images have volumetric and texture details and it is impossible to obtain such details with conventional microscopes.

The technology enables to capture thin optical sections from thick specimens with controllable depth of field to produce 2D z-stack images through a three-dimensional (3D) object with accurate information, allowing 3D reconstructions to be generated with the digital data from 2D z-stack images. Processing turns 2D images into a 3D image [1, 2], revealing latent features of specimens from carious lesions and inflamed gingival tissues.

Confocal microscopy is a widely applied tool for studying the functions and activities of live cells through a time-change, dye diffusion, and concentration of fluorescent-labeled substances, and also studying for the cellular functions of drug applicants. For live cell imaging, confocal optics provides a major improvement in dimensional resolution and real time [4].

Most microscopic samples are essentially transparent, and the depths of fields of focused samples are exceptionally narrow. Therefore, another advantage of confocal laser scanning microscope (CLSM) is the capacity to distinguish between different depths of a sample, which is free of out-of-focus blur for thin or thick specimens [1]. This process can provide good quality high resolution images for publishing.

In this vitro study, overviews of four applications for 3D/4D microscopic imaging and processing in clinical dental research were introduced. We utilised the Fluoview 1000 Olympus confocal system to study 3D reconstruction for thick sections (up to 100 μm) of dental pulp, 3D reconstruction for oral pathogens in periodontitis tissues, 4D imaging and processing of live cells including time (XYZT) or position (XYZP) to assess proteins of oral pathogen, and confocal microscopy application in drug development.

## Methods

### Carious teeth

The detailed processing of teeth has been described in the previous paper [5]. Briefly, healthy (*n* = 15) and carious teeth (*n* = 37) were obtained with approval of the Ethics Committee of Sydney West Local Health Service and informed consent from patients, aged from 20 to 45 years. The half tooth contained the pulp was fixed in 1 % paraformaldehyde in PBS (phosphate-buffered saline) for 3 h, then washed in PBS and EDTA (ethylenediaminetetraacetic acid, pH 7.0) for 5 or 6 days, changed EDTA each day and equilibrated in 30 % sucrose (*w/v*) for 3 to 5 days at 4 °C. A diamond disc (Thin-Flex, Abrasive Technology, Chicago, IL) was used to trim enamel and the majority of dentin. Samples were cooled down with ample water. Specimens were in cryo-embedding matrix -Tissue Freezing Medium (Triangle Biomedical Sciences, Durham, NC) for quick chill (5 min), and frozen in liquid nitrogen. Sections of 40–100 μm were prepared and stored at −80 °C until required.

### Gingival tissues

The detailed processing of gingival tissue specimens has been described in the previous paper [6]. Briefly, obtained gingival tissues were approved by the Ethics Committee of Sydney Dental Hospital and informed consent from adult participants (*n* = 26) in the periodontal clinics. All patients had detailed clinical records and radiographs with no systemic disease and no periodontal therapy for the past 3 years. Tissues were grouped by clinical and histological criteria as clinically healthy gingival sites and paired periodontitis sites. Gingival tissues were snap-frozen in isopentane, cooled in liquid nitrogen, and 20–40 μm sections prepared for study.

### Oral epithelial cell culture for live/fixed cell imaging

The H413 epithelial cell line original from a human oral squamous cell carcinoma [7] exhibits stratified epithelial cell morphology and high CD24 marker expression in culture. Cell clonal lines of H413 were constructed using a limit dilution method as described previously [8]. H413 colone-1 cells were cultured in Joklik modification’s minimum essential medium (Sigma-Aldrich), supplemented with penicillin/streptomycin (100 IU/ml, Sigma) and 10 % fetal calf serum (FCS, CSL Limited, Victoria, Australia) at 37 °C in 5 % CO_2_ [9]. Cultures were collected with trypsin replacement - triple express (Invitrogen, Australia) in PBS and sub-cultured every 3 days.

To further confirm biological function, live cell imaging was performed to determine intracellular versus paracellular pathways of movement of labeled dextran. Cloned H413-1 cells (2 × 10^5^/cm^2^) were cultured in 8-well slide chambers (ibidi, cat 80826, Germany), 300 μl per well. Confluence was achieved within 48 h. A haemolytic gingipain adhesin domain K2 (100 pM) from *Porphyromonas gingivalis* [10] or a gingipain haemagglutinin sub-domain named Ka 100 pM (constructed in our laboratory, unpublished) was added and each well was simultaneously supplemented with a low molecular weight dextran Alexa Fluor 647 (10 kDa, Invitrogen, Australia) at 1:50 dilution from a stock solution of 1 mg/ml in medium.

For drug development assay, oral pathogen *P.gingivalis* strain ATCC 33277 at MOI (multiplicity of infection) 100 cells per one epithelial cell [11] was added to confluent H413 clone-1 epithelial monolayers for 1.5 h at 37 °C in 5 % CO_2_. Monolayers were washed twice with Dulbecco’s phosphate-buffered saline (DPBS) [12]. Gentamycin (300 μg/ml, Sigma) and metronidazole (200 μg/ml, Sigma) were added to kill adherent *P.gingivalis* on the cell surface (extracellular bacteria, [12]). After an additional 1 h incubation and washing off antibiotics, we added a porphyrin-linked metronidazole adduct (40 μM) developed in our laboratory (auto-fluorescence in red, [13]) to cell culture up to additional 1.5 h. Then *P. gingivalis* was targeted by mouse monoclonal antibody IIB2 (5 μm/ml) [14] at various time points. For live cells, nuclei were stained with NucBlue (Invitrogen, Australia). For fixed cells, the secondary antibody was goat anti-mouse conjugated with Alexa Fluor 488 (Life Technologies, USA).

### Immunostaining for frozen sections

Frozen sections including teeth and gingival tissues were fixed in 4 % paraformaldehyde in PBS for 30 min at room temperature. Slides were washed with PBS and placed in glycine-PBS for 10 min, then washed in PBS and incubated in PBS containing 0.2 % Tween-20 and 10 % goat serum for 1 h at room temperature. Sections were washed in PBS and incubated with primary antibodies: polyclonal rabbit anti-human GFAP (5 μg/ml, Dako), polyclonal rabbit anti-human S100 (4 μg/ml, reacts strongly with human S100B, Dako), polyclonal rabbit anti-human amelogenin (5 mg/ml, Abcam, UK), and mouse monoclonal antibody IIB2 (5 μm/ml) [14] to *P. gingivalis* bacteria, for 1 h at room temperature. For controls, rabbit or mouse IgG (Dakocytomation) was served as the primary antibody. Sections were washed 3 times in PBS and then incubated with secondary antibodies: goat anti-rabbit or mouse IgG conjugated with Alexa Fluor 488 or 594 (Life Technologies, USA) for 1 h at room temperature. Slides were mounted in Prolong gold anti-fade reagent with DAPI (Molecular Probes, Invitrogen).

### Confocal laser scanning microscopy

An Olympus Fluoview (FV) 1000 was used to capture confocal images under multi lasers (405 nm, 473 nm, 633 nm) and NTT electronic Optiλ (559 nm). Visual observation under the objective lens [Olympus 40X/1.30/0.20 (WD) Oil UPLSAPO] was performed. The cells were selected at random and adjusted focus before image acquisition.

For 3D reconstruction imaging on thick teeth and gingival tissue sections, the fundamental step was in staining of the preparations. An appropriate stained section showed the same intensity from the top to the bottom of the stack. Over-stained or under-stained sections would greatly reduce imaging clarity of thick teeth and gingival tissues. Z-stack imaging was performed with a 40X objective using up to three corresponding lasers (405 nm, 473 nm and 559 nm). Briefly, the procedure was to set up step size between 0.5 and 1.5 μm depending on the thickness of samples, and then consecutive cross-section images (XYZ) were acquired from the top to the bottom. After acquisition, projection of z-stack images was displayed using the click Z mode. 3D reconstruction and display of cubic imaging were built up by 3D Olympus Fluoview software. It was essential for 3D reconstructions achieved identical to any z-stack preparation [15].

For 4D (3D time lapse) imaging on live cells, chamber slides with stained samples were operated in the microscope stage incubation chamber (with temperature controller at 37 °C and CO_2_ controller at 5 % of CO_2_) [16]. Time-lapse imaging was performed over a period of hours with a 40X objective using the time-lapse function of confocal microscopy Auto Imaging System. Two corresponding lasers (405 nm and 633 nm) were selected. Combined z-stack and time-lapse functions were used in the present study to swiftly capture the images [16]. Serial images were taken at regular time points to capture the dynamics.

All fluorescence images captured with confocal acquisition software (FV10-ASW 1.7) were stored as Olympus Image Format (OIF) for signal analysis and exported images as TIF files.

### Imaging analysis

Three-dimensional (3D) reconstruction images were processed using Olympus Fluoview (FV) software (4.2 viewer, Japan) or Image J software (version 1.50). Co-localization image analysis was as described in reference papers [17, 18] or by using recently developed advanced software: Huygens Professional (https://svi.nl/HuygensProfessional).

## Results

### Three-dimensional reconstruction for thick carious lesions

**Fig. 1:**
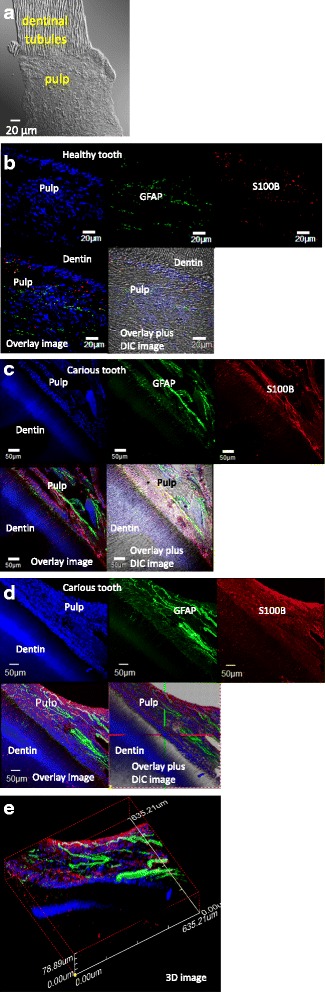
**a** Differential interference contrast (DIC) image displayed the anatomical structure of hard tissue dentin connected with soft pulp tissue from a tooth. **b** 2D images of a healthy control tooth showed scant expression of glial markers GFAP (*green*), S100B (*red*) and the overlay image with DIC channel to show the tooth orientation. **c** A matched set of 2D images showed increased expression of glial markers GFAP (*green*), S100B (*red*) and the overlay image with DIC channel for the orientation of an adult carious tooth. **d** Projection of 2D z-stack images of an adult carious tooth. **e** A 3D image generated by 2D z-stack data (**d**) from a thick carious specimen (78.89 μm)

Specimens used in this study were soft pulp tissues connected with hard dentin tissues (Fig. 1a). 2D images with an overlay of the differential interference contrast (DIC) channel image of a healthy control tooth were displayed scant expression of GFAP and S100B (Fig. 1b). For comparison, a matched set of 2D images from a carious tooth was shown in Fig. 1c. Figure 1d showed projection of 2D stack images from a carious tooth (Fig. 1c) using frozen (40–100 μm) sections, 40–50 images of 0.8–1.5 μm step size acquired as z-stacks by confocal laser scanning microscopy. Three-dimensional (3D) imaging from 2D z-stack images (Fig. 1d) was reconstructed and viewed using confocal software (4.2 viewer, Japan) (Fig. 1e). A 3D movie in the Additional file 1: Movie 1 revealed latent features of glial cell markers GFAP (green) and S100B (red) from thick specimens.

**Fig. 2:**
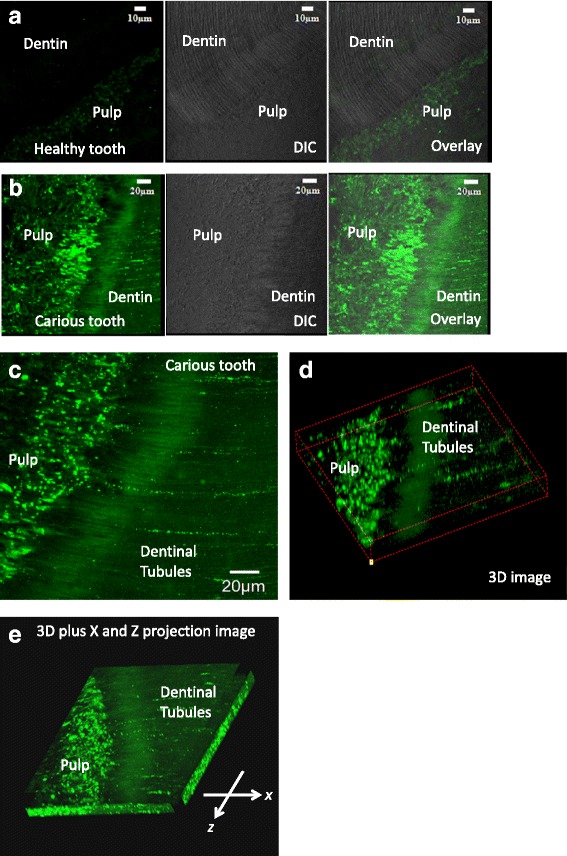
Another example of amelogenin protein strongly re-expressed in newly differentiated pulp cells (odontoblasts) and distributed in dentinal tubules under the lesion site. **a** 2D images of a healthy control tooth, **b** a matched set of 2D images was from an adult carious lesion. **c** Projection of 2D z-stack images from a carious tooth, **d** a 3D reconstruction image from (**c**)﻿, **e** a 3D image plus x and z axes projection

Another example was distribution of amelogenin protein (green) expressed in carious adult human teeth although not detected in healthy adult human teeth. Figure 2a showed 2D images of a healthy control tooth displaying trace expression of amelogenin protein considered as background staining compared to isotype control antibody staining (data not shown). An overlay image using the DIC (differential interference contrast) channel showed anatomical structure and orientation of the tooth. For comparison, a matched set of 2D images from a tooth with a carious lesion was shown (Fig. 2b). Figure 2c showed strong expression of amelogenin protein in pulp cells (odontoblasts) and in dentinal tubules in the projection of 2D z-stack images, Fig. 2d﻿ and e displayed 3D images built from 2D z-stack images (Fig. 2c), and a 3D movie was shown in the Additional file 1: Movie 2.

### Three-dimensional reconstruction for detection of an oral pathogen in periodontitis tissues

**Fig. 3:**
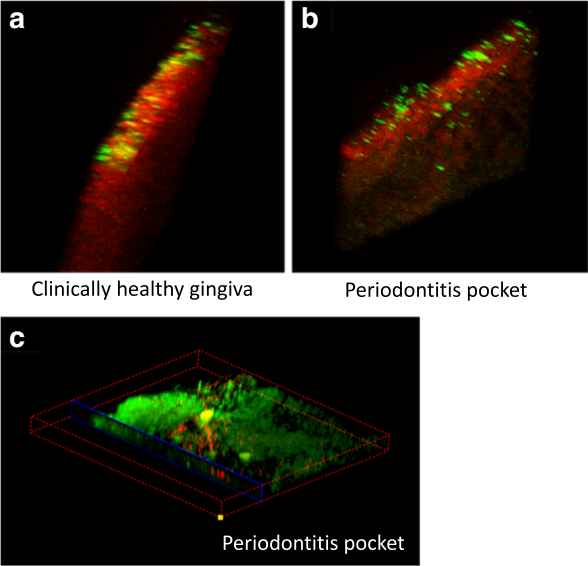
Example of 3D reconstruction to analyse oral microbial biofilm structure. One of the specific pathogens *P. gingivalis* (*green colour*) was found to be widely localised on the surface of clinically healthy gingiva (**a**), but inside the periodontal pocket epithelial tissue from a series of 2D z-stack images (**b**) and one of 3D cross cutting sections, *P. gingivalis *(*red colour*) (**c**)

Using 3D reconstruction to analyse oral microbial biofilm structure, we found one of the specific pathogens *P. gingivalis* to be widely localised inside the periodontal pocket epithelial tissues from a series of 2D-stack images from periodontal disease tissue samples (Fig. 3b) compared to clinically healthy gingiva where the pathogens were only located on the gingival surface (Fig. 3a). The image in Fig. 3c showed a cross cutting section by confocal software, contained *P. gingivalis* (red) inside pocket epithelium. The 3D cross cutting section movie was shown in the Additional file 1: Movie 3.

### Four-dimensional imaging and processing on live cells to assess oral pathogen proteins

**Fig. 4:**
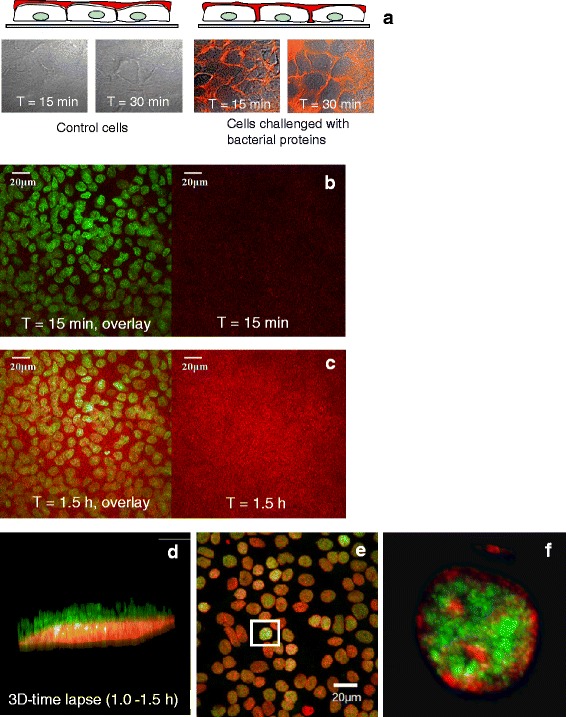
**a** A model of adding oral pathogen *P. gingivalis* proteins K2 and fluorescent dye (Alexa Fluor dextran 647, *red colour*) to confluent gingival epithelial monolayers, to track pathological changes of epithelial integrity at a time course. Paracellular pathway of movement of the labelled dextran (*red*) was shown at 15 and 30 min observation times. **b** and **c** 2D z-stacking images in the beginning (T = 15 min) and end of the exposure time-lapse period (T **=** 1.5 h): left panels were the overlay images of cells (*green nuclei*) with far-red dye channel; right panels were far-red dye channel only. **d** 3D reconstruction time lapse images from (**b-c**) on live cells challenged with bacterial protein (K2) (*green colour*: cell nuclei; *red colour*: dye). **e** Another example of projection of 2D z-stack imaging plus a position point. **f** A 3D reconstruction image with a position point (**e**) on live cells challenged with *P. gingivalis* bacterial protein (Ka) at 30 min (*green colour*: Ka protein; *red colour*: nuclei)

Confocal microscopy used on live cells through time-change, dye diffusion, and concentration of fluorescent-labeled substances. In this study, we added the oral pathogen protein domain K2 from *P. gingivalis* constructed in our laboratory [10] to confluent gingival epithelial monolayers from cell culture, then added fluorescent dye (dextran Alexa Fluor 647) to measure the diffusion patterns of fluorescent molecules in response to this bacterial virulence factor, which attacked epithelial integrity. Figure 4a showed a model of paracellular movement of the labelled dextran (red) for cells challenged with K2 at 15 and 30 min observation times. Figure 4b and c showed 2D z-stacking time lapse images in the beginning (T = 15 min) and end of the exposure time-lapse period (T **=** 1.5 h) for cells challenged with K2. Figure 4d showed 3D reconstruction time lapse image (XYZT, 4D) when including time points, and a 4D movie was shown in the Additional file 1: Movie 4.

Another example of 4D image processing including position points (XYZP). Figure 4e showed 2D z-stack image in which *P. gingivalis* proteins Ka (green) penetrated into nuclei (red) after 30 min on live cells. One of the cells was picked from 2D z-stack images to build up a 3D image (XYZ Plus position point, Fig. 4f). A 4D movie was shown in the Additional file 1: Movie 5.

### Confocal laser scanning microscopy in drug development

**Fig. 5:**
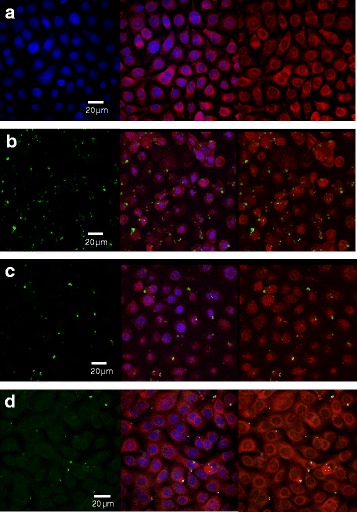
**a** A pathogen-specific antimicrobial compound (a porphyrin-linked metronidazole) developed in our laboratory [13] which can target intracellular oral pathogens *P.gingivalis* displayed in red auto-fluorescence. Left: nuclei staining with DAPI (*blue channel*), middle: an overlay image of drug (*red channel*) with nuclei (*blue channel*), and right image was drug only (*red channel*). Images showed that this modified drug penetrated into the cells and localized within the cytoplasm (*red*) to kill oral intracellular pathogens *P. gingivalis* (*green*) with a time course at 30 min (**b**), 1 h (**c**) and 1.5 h (**d**). From (**b**–**d**), left panels showed green colour bacteria *P. gingivalis*; middle panels showed the overlay images of cells^'^ nuclei (*blue channel*), *P.gingivalis* (*green channel*) and drug (*red channel*); right panels showed the overlay images of *P.gingivalis* (*green channel*) and drug (*red channel*) only

Using confocal microscopy, it was possible to identify the penetration/localization/co-localisation of a porphyrin-linked metronidazole antibiotic and *P. gingivalis* bacteria. The porphyrin adducts exhibiting auto-fluorescence in the red range of wavelengths (600–650 nm), penetrated into the cells and localized within the cytoplasm (Fig. 5a). Immuno-stained *P. gingivalis* potent gingipain-RgpA was shown in Fig. 5b–d in green fluorescence, also localized within cytoplasm. This modified drug could kill oral intracellular pathogens *P. gingivalis* with a time course at 30 min (Fig. 5b), 1 h (Fig. 5c) and 1.5 h (Fig. 5d).

## Discussion

Why do we need confocal microscopy as a visual tool for clinical dental research? As noted previously, confocal microscopy has several advantages particularly for 3D visualization on thick specimens to reveal latent features of cell and tissue structures. The present study demonstrates that latent patterns of the increased glial cell markers GFAP and S100B on carious teeth lesions can be revealed through 3D reconstruction from thick specimens. The increased abundance of these markers in carious teeth lesions indicates a response to initial microbial invasion of dentin [5]. In the current study, 3D reconstruction also reveals strong expression of amelogenin protein in pulp cells (odontoblasts) and in dentinal tubules from adult human teeth with carious lesions. In a normal condition, this amelogenin protein is only appeared during tooth embryonic development including expression in tooth enamel, dentin, and pulp cells (odontoblasts) [19]. When tooth becomes mature, amelogenin is absent in dentin and pulp cells (odontoblasts). However, in injured or carious teeth amelogenin protein is highly re-presented in newly differentiated pulp cells (odontoblasts) and allotted in the dentinal tubules under the adult carious teeth [19] corresponding to our findings.

Dental researchers face major issues when cutting thin sections of soft pulp tissues connected with hard dentin tissues. Therefore, we utilise the advantage of analysis of fluorescent labeled thick specimens without physical sectioning for carious teeth and thick gingival tissues.

Confocal laser scanning microscopy (CLSM) is a useful method to study the interface of bacteria within biofilms and inflamed gingival tissues. Images obtained from CLSM are free of out-of-focus blur. CLSM-based imaging platform has been widely used for analysis of oral bacterial pathogens or biofilm structures on gingival tissues [20–23]. The images shown in the present study illustrate that 3D visualization is a promising method for representative analysis of oral bacterial pathogens or biofilm components [20].

In the present confocal microscopy study, using 4D (3D time lapse) imaging and processing on live cells we demonstrate that time-lapse imaging is a powerful technique for analysis of dynamic cellular events and morphology in real time and real space. There are two issues that researchers need to be aware of while performing experiments on live cells. Firstly, how to choose the right dye? The excitation (absorption, short wavelength) and emission (long wavelength) spectra of the Alexa Fluor series cover the visible spectrum and extend to the invisible infrared [24]. The closer to infrared wavelengths, the less damage to live cells [25], therefore, the far-red dye of Alexa Fluor 647 was chosen in the current study.

Secondly, illumination by fluorophores can cause photo bleaching and cell damage, hence there is a need to raise the speed of image acquisition by confocal microscopy. However the confocal microscopy has a limitation of raising an acquisition speed, which can result in a low resolution of image [26]. This image resolution can be improved by the use of DeltaVision super-resolution microscope (GE Healthcare, Japan). DeltaVision microscopy can offer better performance at high magnification (100 times), absolutely high resolution and contrast through de-convolution of images, especially on live cell imaging where faster image acquisition, lower excitation power, and less photo bleaching and cell damage [27, 28]. This is a key consideration which can be improved by DeltaVision microscopy in the future study on live cells [27, 28].

Using confocal microscopy in the present drug development study, has determined that a novel porphyrin-linked metronidazole antibiotic can penetrate into epithelial cells to kill intracellular oral pathogens. However, researchers need to be aware of that an overlap in fluorescence does not necessarily demonstrate co-localization of two colours (probes) in the same cellular structure [17]. Therefore the co-distribution of two colours (probes) in fluorescence microscope images can be evaluated quantitatively and statistically on either co-localisation or two colours’ ratio change using Huygens professional software (https://svi.nl/HuygensProfessional). For the images shown in Fig. 5, we could explain it as the ratio change of two colours which bacterial spots in green were decreasing with a time lapse comparing to drugs in red colour.

## Conclusions

Confocal laser scanning microscope is a valuable tool in dental research, particularly for probing thick specimens of carious teeth, formation of microbial biofilms on gingival tissues, tracing pathological changes in live cells and studying the cellular effects of drug candidates. Future developments of powerful microscopy will be of great interest and benefit in clinical dental research.

## Additional file

Additional file 1: Movie 1–5 (PPTX 2493 kb)

## Abbreviations

3D: Three dimension (XYZ)
4D: Four dimension (XYZT or XYZP)
CLSM: Confocal laser scanning microscope
EDTA: Ethylenediaminetetraacetic acid
GFAP: Glial fibrillary acidic protein
*P. gingivalis*: *Porphyromonas gingivalis*
PBS: Phosphate-buffered saline
S100B: S100 calcium-binding protein B
XYZP: 3D (XYZ) plus position points
XYZT: 3D (XYZ) plus time lapse
