# Supplementary material for: Three/four-dimensional (3D/4D) microscopic imaging and processing in clinical dental research
Source: BMC Oral Health. 2016 Sep 1;16(1):84. doi: 10.1186/s12903-016-0282-0 (PMC5009657; doi:10.1186/s12903-016-0282-0)
Supplement: Additional file 1: — Movie 1–5 (PPTX 2493 kb) [file 12903_2016_282_MOESM1_ESM.pptx]

## Slide 1
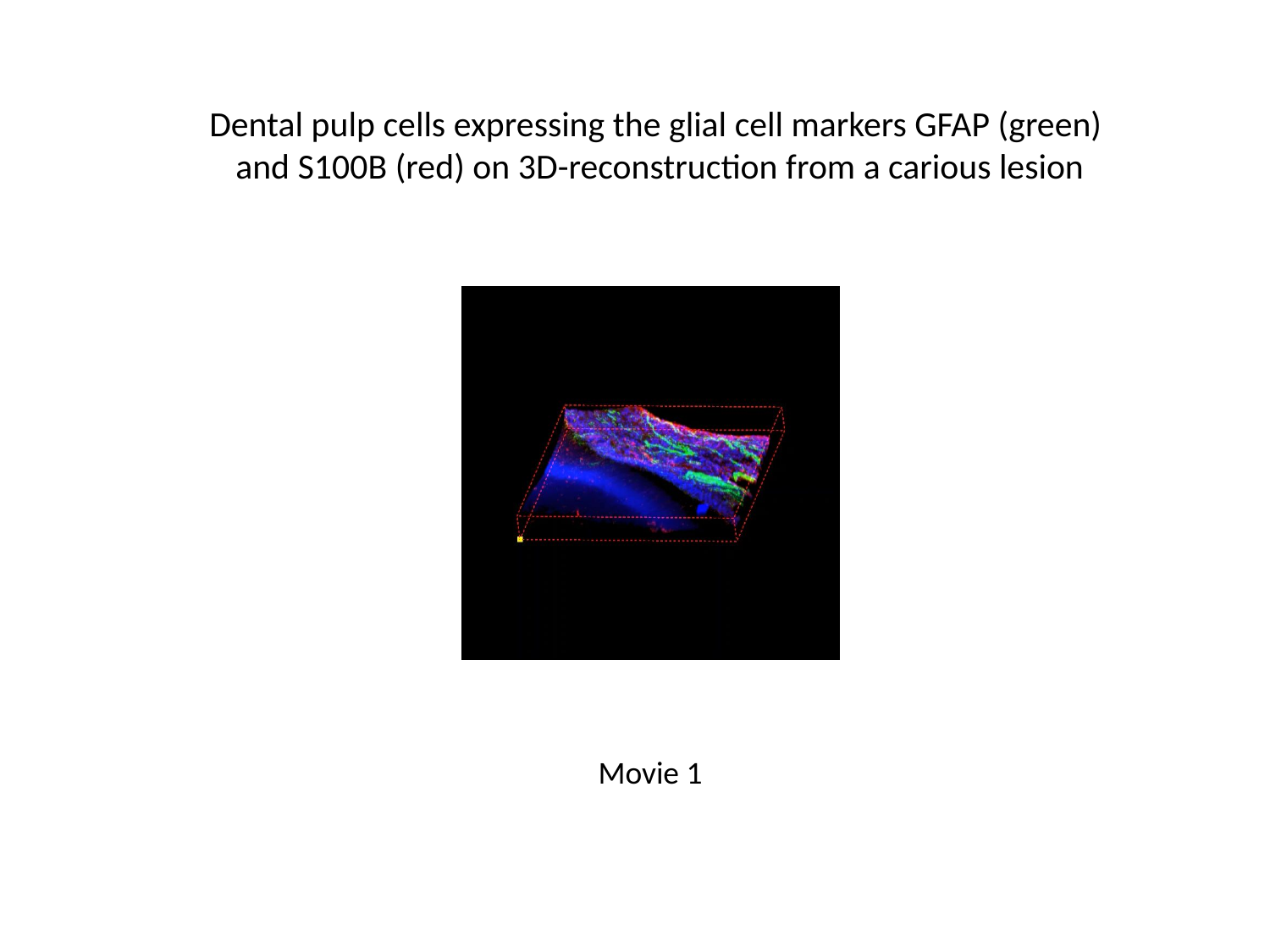

# Dental pulp cells expressing the glial cell markers GFAP (green) and S100B (red) on 3D-reconstruction from a carious lesion
Movie 1

## Slide 2
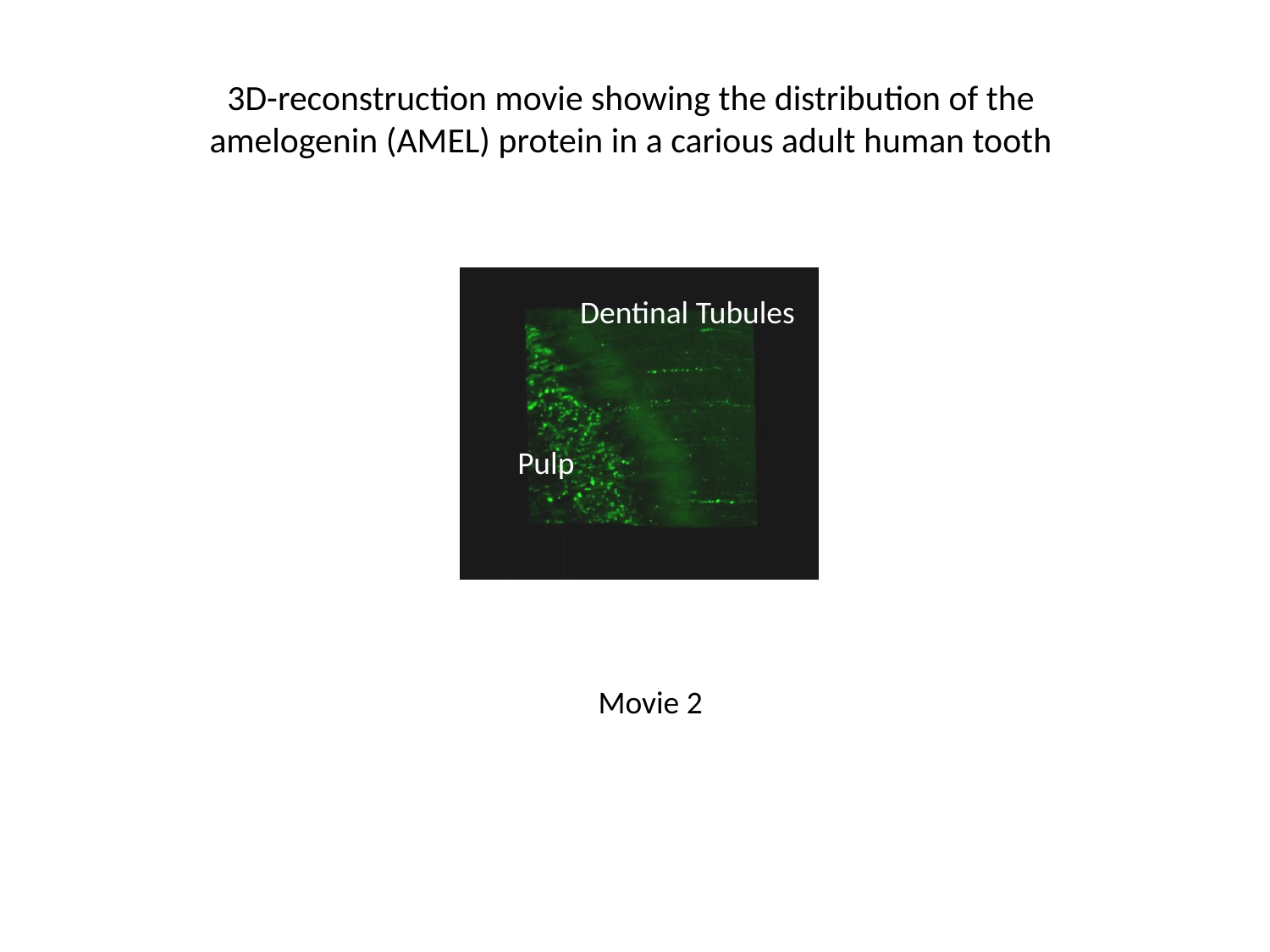

# 3D-reconstruction movie showing the distribution of the amelogenin (AMEL) protein in a carious adult human tooth
Dentinal Tubules
Pulp
Movie 2

## Slide 3
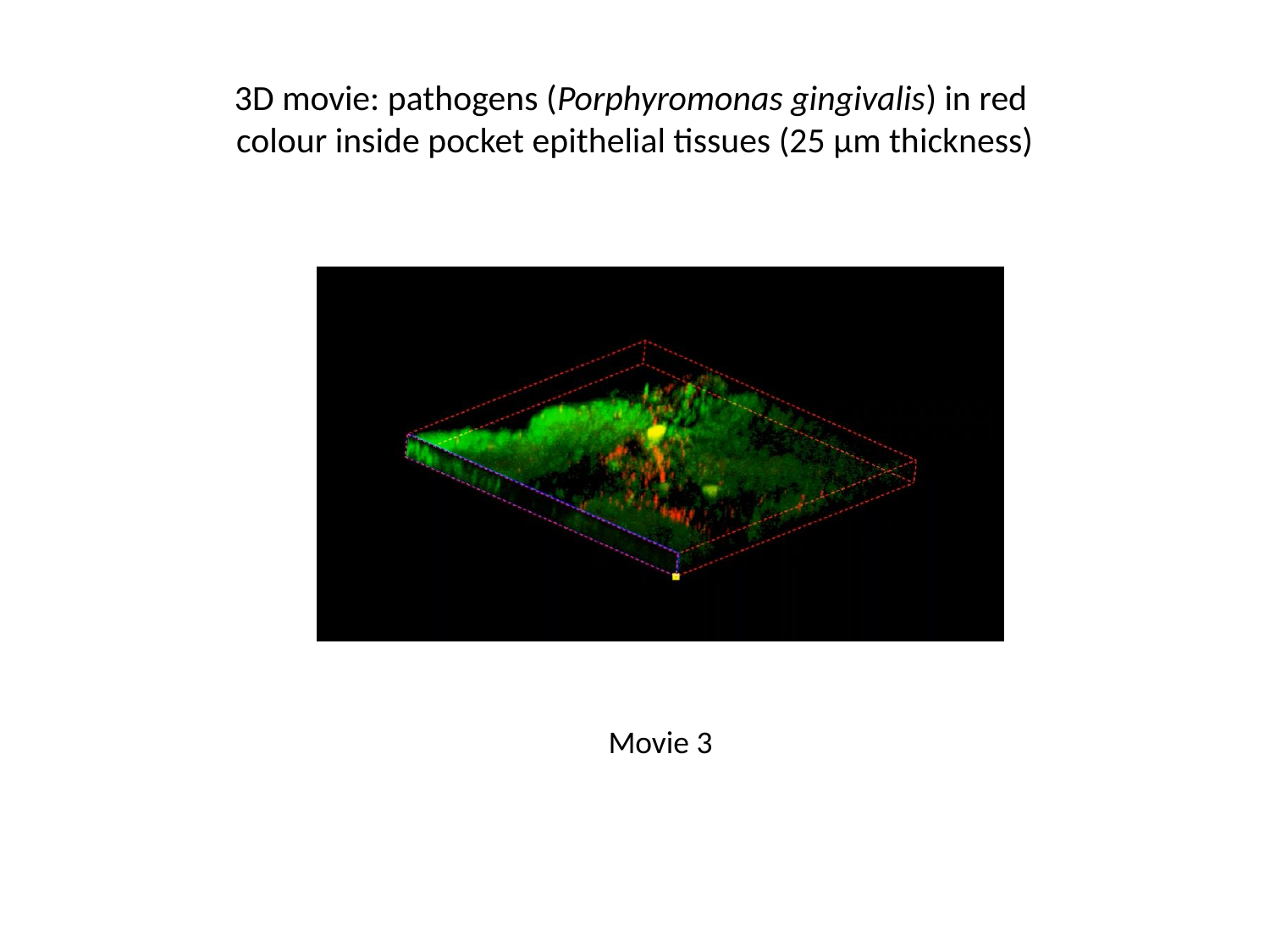

# 3D movie: pathogens (Porphyromonas gingivalis) in red colour inside pocket epithelial tissues (25 µm thickness)
Movie 3

## Slide 4
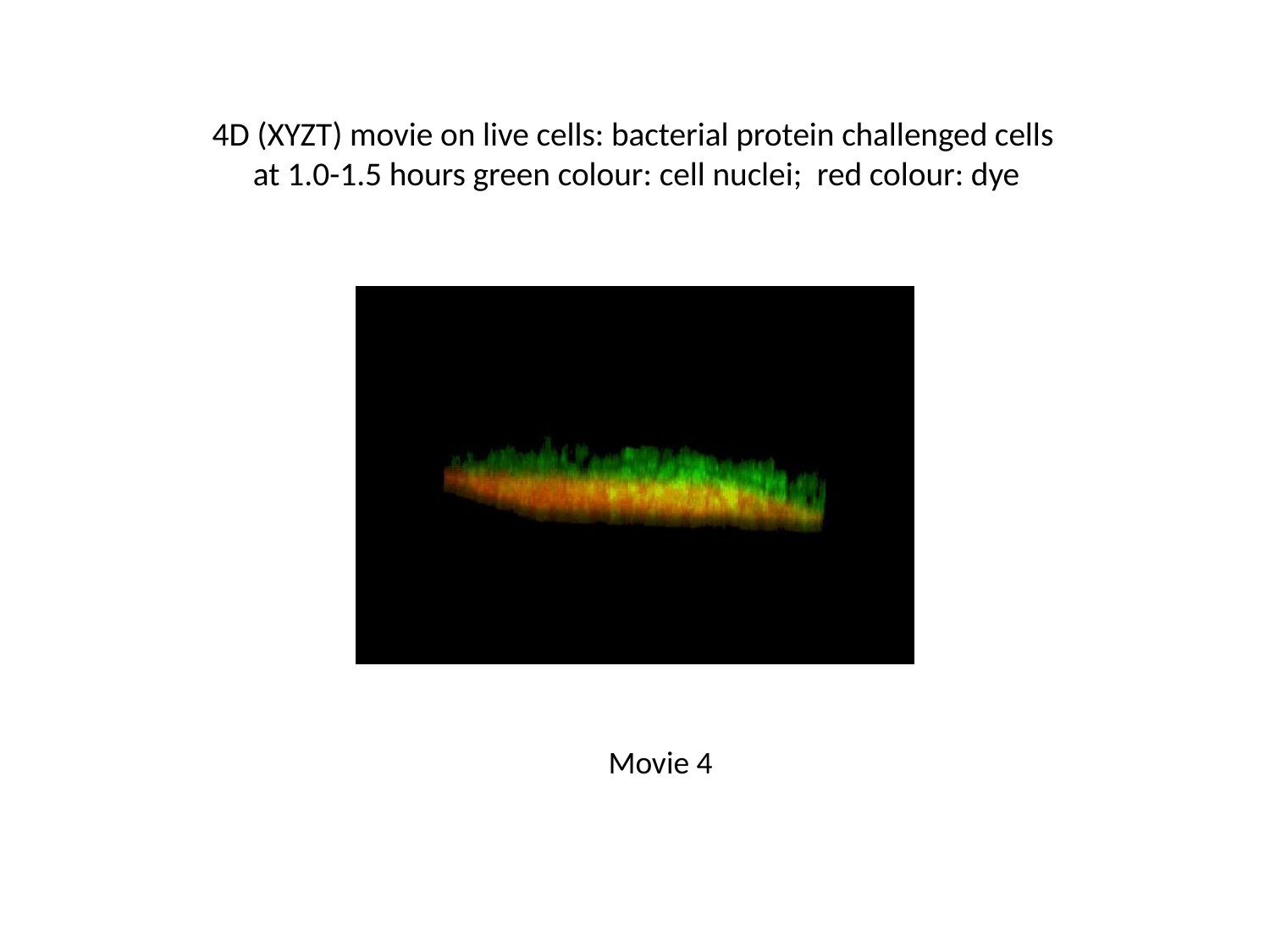

# 4D (XYZT) movie on live cells: bacterial protein challenged cells at 1.0-1.5 hours green colour: cell nuclei; red colour: dye
Movie 4

## Slide 5
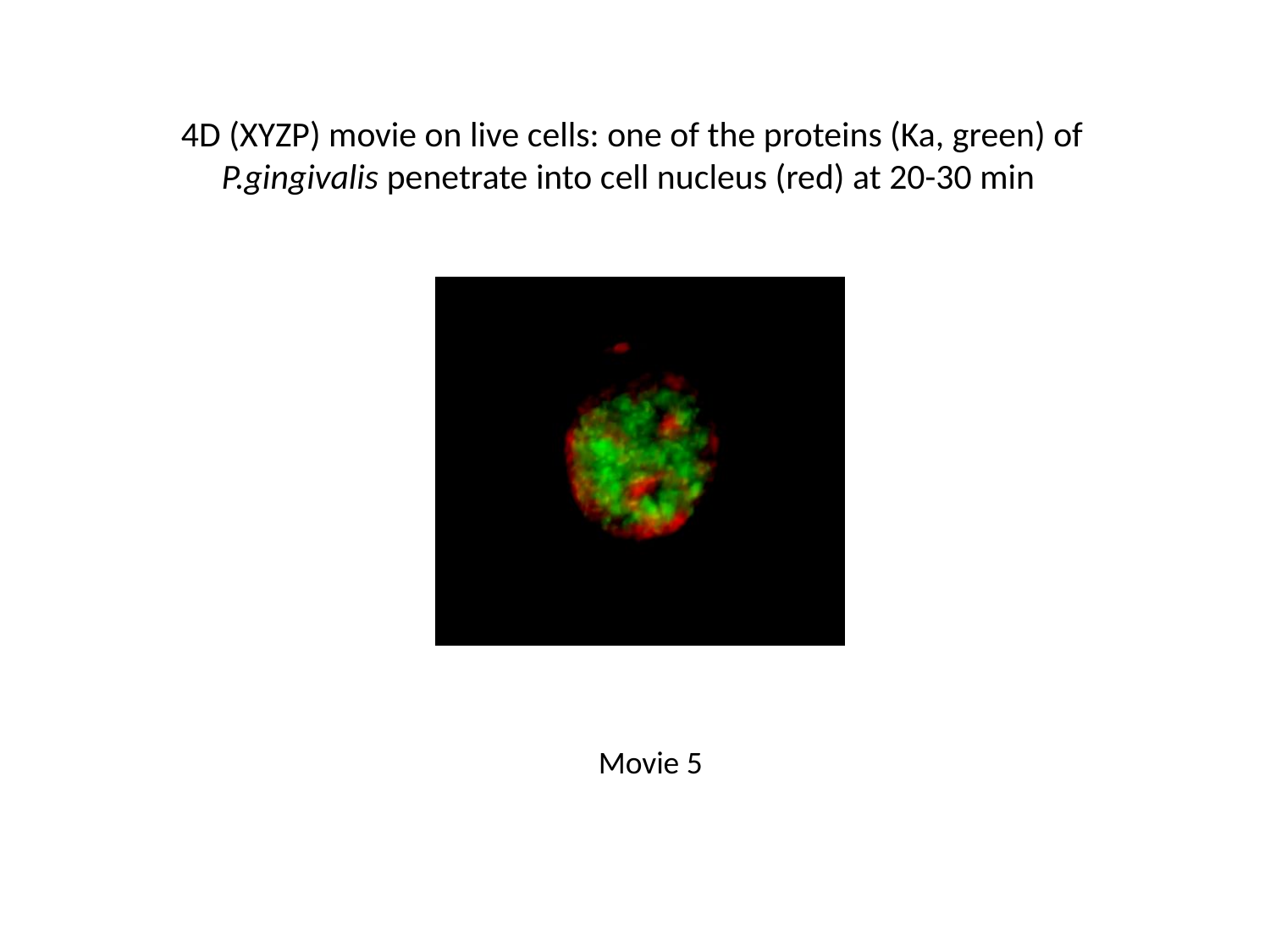

# 4D (XYZP) movie on live cells: one of the proteins (Ka, green) of P.gingivalis penetrate into cell nucleus (red) at 20-30 min
Movie 5
